# Bidirectional interplay between periodontal disease and carotid artery disease: a comprehensive holistic review

**DOI:** 10.3389/fcimb.2025.1747351

**Published:** 2026-01-08

**Authors:** Ana Maria Hofer, Andrei Picos, Alexandra Dădârlat-Pop, Monica Popa

**Affiliations:** 1 Department of Community Health, “Iuliu Hatieganu” University of Medicine and Pharmacy, Cluj-Napoca, Romania; 2 Department of Oral Prevention, “Iuliu Hatieganu” University of Medicine and Pharmacy, Cluj-Napoca, Romania; 3 Cardiology Department, "N Stăncioiu" Heart Institute, Cluj-Napoca, Romania; 4 Department of Internal Medicine, Faculty of Medicine, "Iuliu Hațieganu" University of Medicine and Pharmacy, Cluj-Napoca, Romania

**Keywords:** atherosclerosis, cardiovascular risk, carotid artery disease, pathogen, periodontal disease

## Abstract

**Background:**

Periodontal disease and atherosclerosis are two highly prevalent chronic inflammatory conditions that may be pathophysiologically linked.

**Objective:**

This review aims to critically assess the current literature regarding the potential causal relationship between periodontitis, the atherosclerotic process, and particularly the progression of carotid artery disease. Although numerous observational studies support an association between periodontitis and cardiovascular diseases, the evidence remains inconclusive in demonstrating periodontal disease as an independent risk factor in the development of carotid atherosclerotic plaques.

**Conclusion:**

While there is substantial biological plausibility for a link between periodontal disease and atherosclerosis, current evidence is insufficient to confirm a direct causal role, particularly in carotid artery disease. Further longitudinal and interventional studies are necessary to elucidate the mechanisms involved and determine whether periodontal treatment may contribute to cardiovascular risk reduction. The potential public health implications of these findings highlight the necessity for comprehensive preventive and therapeutic oral health interventions aimed at reducing associated systemic risks.

## 1 Introduction

Periodontitis is a common chronic inflammatory disease that irreversibly affects the tooth-supporting structures. It is characterized by gingival bleeding, loss of clinical attachment, and resorption of alveolar bone, indicating inflammation and progressive tissue destruction (Chen et al., 2021). Beyond its impact on oral health, periodontitis is increasingly recognized as a potential risk factor for various systemic conditions, affecting approximately 20% to 50% of the global population (Chen et al., 2021). Moreover, severe periodontitis is estimated to affect about 9% of the global population, making it the tenth most prevalent noncommunicable disease worldwide (Global Burden of Disease Study 2013 Collaborators, 2015).

## 2 Periodontitis risk factors

Risk factors for periodontitis can be broadly categorized into non-modifiable and modifiable factors (Makkar et al., 2018). Non-modifiable risk factors include age, ethnicity, genetic predisposition, and male gender (Makkar et al., 2018). There is strong evidence showing that the prevalence and severity of periodontitis increase with age. Additionally, men present a 50% higher risk of periodontitis in comparison with women (Makkar et al., 2018).

Modifiable risk factors—such as diabetes mellitus (DM), smoking, alcohol consumption, sedentary lifestyle, stress, —also play significant roles in the development and progression of periodontitis (Makkar et al., 2018). These risk factors are shared with those for cardiovascular diseases, which may serve as a potential argument supporting the association between the two pathologies.

Smoking is one of the most well-established periodontitis risk factors, with smokers having four to five times higher odds of developing periodontitis compared to non-smokers (Makkar et al., 2018). A dose-dependent relationship has also been observed. Also, it ranks as the fourth most significant attributable risk factor for ischemic heart disease in men and the seventh in women (Dai et al., 2022). Smoking cessation interventions remain among the most important preventive strategies for both cardiovascular and periodontal disease.

While establishing a direct causal relationship between periodontitis and atherosclerotic cardiovascular disease (ASCVD) remains challenging, recent studies suggest that improved management of periodontal disease may contribute to a reduced risk of ASCVD (Carra et al., 2023). Despite ongoing debate and conflicting evidence, numerous investigations have explored the nature of this association.

At the 2012 workshop jointly organized by the European Federation of Periodontology and the American Academy of Periodontology, the available evidence on the association between cardiovascular and periodontal diseases was thoroughly reviewed. The discussions focused on two key aspects: the role of bacterial invasion and infection, and the contribution of inflammatory mechanisms to disease progression (Herrera et al., 2020).

## 3 Periodontal pathogens

### 3.1 Oral microbioma

The oral cavity hosts a highly diverse microbiological eco-system, shaped by constantly changing the mechanical and chemical conditions. These dynamic fluctuations create multiple ecological niches that support the proliferation of a wide range of oral microorganisms. To date, more than 700 microbial species have been identified within the oral environment, of which approximately 500 are bacterial species (Raizada et al., 2017). The predominant bacteria belong to several major phyla, including Actinobacteria, Proteobacteria, Firmicutes, Bacteroidetes, Tenericutes, Euryarchaeota, Chlamydiae, and Spirochaetes (Reynolds-Campbell et al., 2017).

Periodontal disease arises when the equilibrium between the host and the microbial community is disrupted, a phenomenon known as dysbiosis. The balance of this relationship can be disturbed by an unusually high microbial burden or by an improper host immune response (Cugini et al., 2021).

Distinct regions of the oral cavity are densely populated with commensals whose composition changes from birth to old age in response to factors such as salivary flow, tissue condition, hormones, and diet. After tooth brushing, a well-defined sequence of colonization begins on the enamel surface (Socransky and Manganiello, 1971). Pioneer Gram-positive bacteria first attach to the salivary pellicle, forming organized arrays on the tooth surface (Kolenbrander et al., 2006; Esberg et al., 2020). These are followed by secondary and tertiary colonizers, all commensal residents of the oral microbiota (Lane, 2015).

The microbial load in gingivitis is increased (10^4^–10^5^ bacteria can be cultivated from a single pocket), being associated with an increase in the percentage of Gram-negative bacteria (15–50%) (Darveau et al., 1997). A greater presence of periodontopathogens is also noted.

The microbial load in periodontitis increases even more (10^5^–10^8^ bacteria can be cultivated from a single pocket), of which approximately 70% are Gram-negative. The presence of periodontopathogenic bacteria is evident: Tannerella forsythia, Porphyromonas gingivalis, Treponema denticola, Prevotella intermedia (Socransky and Manganiello, 1971), which group together in greater numbers in diseased sites. Other, less virulent species may also be present: Eikenella corrodens, F. nucleatum, Capnocytophaga spp, Parvimonas micra (Ximénez-Fyvie et al., 2000).

Metatranscriptomic analysis of the subgingival biofilm associated with periodontitis has revealed the activity of the entire dysbiotic community, not only that of periodontal pathogens. Thus, an active transcription of virulence factors was identified for both categories of bacteria (Marsh and Zaura, 2017).

### 3.2 Specific bacterial complexes

The notion of a bacterial complex is supported by the observation that when the presence of subgingival bacteria is detected, there are frequently very strong associations among certain groups. These observations have led to the description of microbial complexes with similar pathogenic potential. The presence of these specific subgingival bacterial complexes can be associated with certain stages of periodontal disease progression (Haffajee et al., 1998; Socransky et al., 1998).

The red complex is represented by *Tannerella forsythia, Porphyromonas gingivalis, and Treponema denticola* (**Table 1**). This complex has been associated with deep periodontal pockets and areas of clinical attachment loss. Species from the orange complex (**Table 1**) may be found in over 90% of samples. Species of the red complex are most often associated with sites exhibiting clinical signs of disease, while those of the orange complex seem to precede colonization by red-complex bacteria (Haffajee et al., 1998; Socransky et al., 1998; Armitage, 2004). Majority of bacteria belonging to the violet, yellow, and green complexes are associated with periodontal health (Charon and Mouton, 2003a; Brochut et al., 2005).

**Table 1:** Microbial complexes in subgingival plaque.

Complex	Representative species	Associated with
Red Complex	*Tannerella forsythia; Porphyromonas gingivalis; Treponema denticola*	Periodontitis; deep pockets; attachment loss
Orange Complex	Campylobacter spp. (*C. gracilis, C. rectus, C. showae*); *Eubacterium nodatum*; *Fusobacterium* spp.*; Peptostreptococcus micros* (Parvimonas micra); *Prevotella intermedia; Streptococcus constellatus.*	Disease progression; precedes red complex
Yellow Complex	Actinomyces odontolyticus; Veillonella parvula; Streptococcus gordonii; S. intermedius; S. mitis; S. sanguis	Periodontal health
Green Complex	Capnocytophaga spp.; Eikenella corrodens; early strains of Actinomyces actinomycetemcomitans; Campylobacter concisus; Capnocytophaga sputigena	Periodontal health
Violet Complex	*Streptococcus mitis; Actinomyces naeslundii*	Health; early colonizers
Other newly associated species (not part of classic complexes)	17 newly identified organisms (e.g., from *Bacteroidetes, Firmicutes, Proteobacteria, Spirochaetes, Synergistetes, Candidatus Saccharibacteria*)	Newly linked to periodontitis
Archaea	e.g., Methanobrevibacter oralis	Associated with periodontal disease

Haffajee et al. (1999) analyzed data from the Ximenez-Fyvie et al. (1998) study, which examined 1179 supragingival samples and 1179 subgingival samples from 187 individuals: 47 with gingivitis, 117 with periodontal disease, and 23 periodontally healthy (Haffajee et al., 1998; Ximénez-Fyvie et al., 2000). It was found that both supra- and subgingival plaques associated with periodontal health contain a distinct combination of the previously mentioned complexes. The most significant changes can be observed in the red and orange complexes, which are much more frequently found in subgingival plaque than supragingival plaque. These two complexes together represent 15% of supragingival plaque in periodontal health conditions and much more — 36.5% — in subgingival plaque associated with periodontitis. The increase in the orange complex appears to be due to bacteria of the genus *Actinomyces* (Haffajee et al., 1998).

*Aggregatibacter actinomycetemcomitans* (*A. actinomycetemcomitans*, *A.a*) was first described in 1912 by the German microbiologist Klinger, who isolated it from cervicofacial actinomycosis lesions (Klinger, 1912). Its former designation, *Actinobacillus*, referred both to the star-shaped morphology of its colonies in culture and to the bacillary shape of the bacterial cells. The current species name, *actinomycetemcomitans*, reflects its initial co-identification with *Actinomyces israelii* in actinomycosis lesions (Klinger, 1912).

*A. actinomycetemcomitans* is a Gram-negative, non-motile, capnophilic, facultative anaerobic, saccharolytic coccobacillus. It forms small (0.5–1 mm), strongly adherent colonies on agar that are circular, convex, and translucent, exhibiting a characteristic star-shaped internal structure with irregular margins (Charon and Mouton, 2003b).

This facultatively anaerobic oral pathobiont establishes colonization of the oral mucosa early in life, where it persists as a commensal without inducing host damage (Fine et al., 2019). Subsequently, *A. actinomycetemcomitans* can translocate into the gingival crevice, initiating infections that precipitate destruction of connective tissue, alveolar bone, and ultimately tooth loss (Belibasakis et al., 2019). The species constitutes a major etiological agent of periodontitis in adolescents and young adults (Molli et al., 2023) and is particularly implicated in periodontitis and tooth loss among African adolescents, where its occurrence exhibits familial clustering indicative of a genetic susceptibility to disease development (Belibasakis et al., 2019; Fine et al., 2019). *A. actinomycetemcomitans* displays extensive genetic heterogeneity, with serotypes representing genetically discrete lineages (Yoshida et al., 2021). This heterogeneity yields both benign strains and hypervirulent genotypes—most notably JP2 and cagE—which are characterized by markedly elevated leukotoxin production (Belibasakis et al., 2019; Jensen et al., 2019; Kittichotirat et al., 2022).

The most isolated strains of *A. actinomycetemcomitans* are serotypes a, b, and c. It is important to emphasize that the isolated serotypes mentioned above have different clinical manifestations (Claesson et al., 2020).

Serum resistance is a key virulence determinant, predominantly observed in bacteria that invade the bloodstream and establish infection. This phenotype enables bacterial cells to evade serum-mediated innate immune defenses, including antimicrobial peptides and complement activity (Talapko et al., 2024).

*Aggregatibacter actinomycetemcomitans* is an important member of the HACEK group—along with *Haemophilus influenzae, H. parainfluenzae, H. aphrophilus, H. paraphrophilus, Cardiobacterium hominis, Eikenella corrodens*, and *Kingella kingae*—a collection of Gram-negative bacteria that are difficult to grow because they require special nutrients and specific environmental conditions (Khaledi et al., 2022). Although these organisms normally inhabit the oral cavity and upper respiratory tract, they are also capable of causing various infections, including infective endocarditis (Khaledi et al., 2022). The systemic impact is therefore not limited to individuals carrying *A. actinomycetemcomitans* alone, but rather arises from the interplay between periodontal disease and the presence of this pathogen. Importantly, studies have shown that decreasing the incidence and severity of periodontal disease can lead to a parallel reduction in related systemic conditions (Nazir, 2017).

*Porphyromonas gingivalis* is a Gram-negative, strict anaerobic coccobacillus that forms dark brown to black, non-fluorescent colonies on blood agar enriched with hemin and vitamin K. This bacterium belongs to the Bacteroidaceae family, specifically the group of Bacteroides (Mysak et al., 2014).

Porphyromonas gingivalis was formally recognized as a major periodontopathogen at the 1996 World Workshop in Periodontics (Genco, 1996).

It is one of the most prevalent and numerically dominant species in chronic periodontitis lesions. It is rarely detected in children (Slots and Ting, 1999) but is more frequently isolated from periodontally healthy adults (Slots and Ting, 1999) as well as from lesions of localized aggressive forms of periodontitis.

Its prevalence—alongside *Treponema denticola, Tannerella forsythia*, and *Prevotella intermedia*—increases with age in patients with periodontal disease (Murugaiyan et al., 2024).

The bacterial load of *P. gingivalis* correlates positively with probing depth, active/progressive sites, and sites prone to recurrence (deep periodontal pockets) (Murugaiyan et al., 2024).

The species included in the red and orange complexes are considered only a non-exhaustive list.

Thus, 17 new microbial candidates have been associated with periodontitis, belonging to bacterial phyla already implicated in the disease *(Bacteroides, Firmicutes, Proteobacteria, Spirochaetes, Synergistetes*) or newly identified (*Candidatus Saccharibacteria*). Additionally, members of the domain Archaea have also been associated with periodontal pathogens (Pérez-Chaparro et al., 2014). Microbiological changes in periodontitis reflect not only a disturbance of the microbiota but also the need to understand the structure and function of the microbial population as a whole.

During the transition from periodontal health to disease, pocket depth increases, anaerobic conditions intensify, and the availability of carbon sources declines. Concurrent shifts in inflammation, pH, and temperature further destabilize the semi-delineated ecology of the periodontal pocket (Loesche et al., 1993). Once the epithelial barrier becomes breached or ulcerated, bacteria from the subgingival biofilm can disseminate into the bloodstream. These translocating species, including *Streptococcus, Fusobacteria, Aggregatibacter, Bacteroides, and Porphyromonas*, can establish themselves on compromised vessels or tissues (Brochut et al., 2005; Pérez-Chaparro et al., 2014).

## 4 Microbiological pathways: invasion and infection

### 4.1 Dissemination of periodontal bacteria

Evidence indicates that periodontal bacteria can enter the bloodstream and reach systemic vascular tissues, primarily through transcellular transport or following disruption of the gingival epithelium during dental procedures or routine oral hygiene activities (Takeuchi et al., 2011; Carrion et al., 2012). Furthermore, studies have examined the relationship between periodontal health and the risk of bacteraemia, suggesting that individuals with gingival inflammation are at greater risk (Tomas et al., 2012).

**Figure 1:**
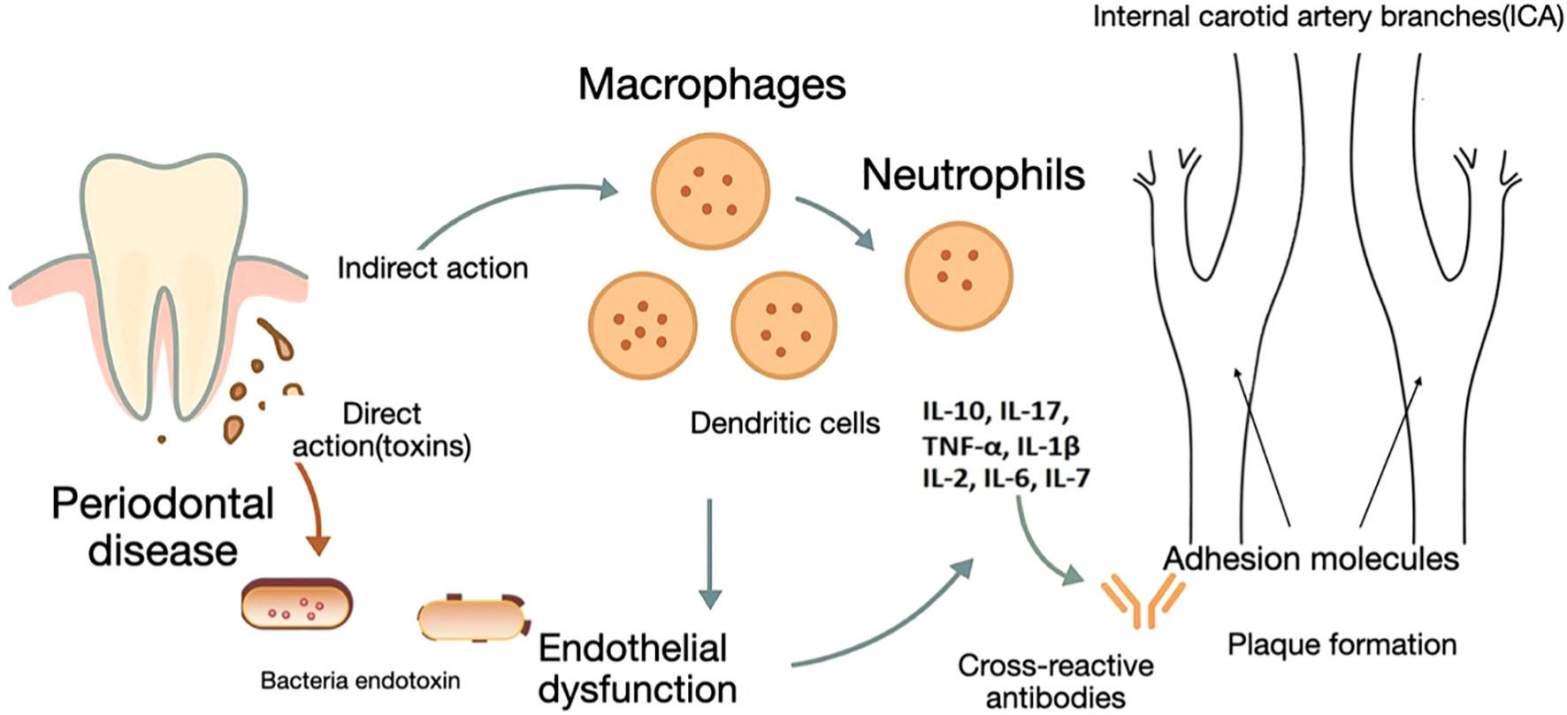
Immune-mediated connection between periodontal disease and carotid artery disease.

### 4.2 Presence of periodontal bacteria in affected tissues

Numerous oral bacterial species have been identified in atheromatous tissues through DNA, RNA, and antigen analyses, often found in combination. Recent advancements such as high-throughput sequencing and cross-sample comparisons have further confirmed that periodontal pathogens can reach and colonizing atheroma plaques. Notably, individuals with periodontitis exhibit a higher risk of harboring these pathogens within vascular lesions (Figuero et al., 2011; Serra e Silva Filho et al., 2014).

### 4.3 Enhanced systemic inflammatory response

Microorganisms rapidly colonize tooth surfaces following oral hygiene procedures. As the microbial load—particularly gram-negative periodontal pathogens—increases within the gingival sulcus, a localized inflammatory response is gradually initiated (Jürgensen and Petersen, 2009; Figuero et al., 2014). This immune response initially manifests as gingivitis, characterized by inflammation confined to the gingiva. However, not all cases of gingivitis progress to periodontitis. In periodontitis, the inflammation extends beyond the gingiva to affect the periodontal ligament and alveolar bone, leading to irreversible tissue damage.

The inflammatory response triggered by the bacterial challenge is not limited to the local periodontal lesion. Elevated levels of cytokines and other inflammatory mediators have been detected in gingival crevicular fluid, saliva, and gingival tissues, indicating a broader systemic involvement. Notably, patients with periodontitis exhibit higher serum concentrations of inflammatory markers compared to periodontally healthy individuals (Ionel et al., 2017).

C-reactive protein (CRP), an acute-phase reactant synthesized by the liver in response to inflammatory cytokines, is frequently elevated in systemic inflammatory conditions, including both chronic and aggressive forms of periodontitis as well as cardiovascular disease (Schenkein and Loos, 2013). Treatment of either condition has been shown to reduce systemic CRP levels. Moreover, individuals suffering from both chronic periodontitis and cardiovascular disease demonstrate significantly higher serum CRP concentrations and polymorphonuclear neutrophil counts compared to those with periodontitis alone or healthy controls (Montero et al., 2020).

Among the various interleukins involved in inflammatory responses, interleukin-1 (IL-1), interleukin-4 (IL-4), interleukin-6 (IL-6), interleukin-8 (IL-8), and interleukin-18 (IL-18) have been extensively studied in periodontal medicine due to their potential shared pathogenic roles in both periodontitis and systemic inflammatory diseases. Notably, patients with moderate chronic periodontitis have been shown to exhibit higher serum IL-6 levels compared to healthy individuals or those with mild disease (Anitha et al., 2013). IL-6 plays a central role in systemic inflammation by stimulating the hepatic production of acute-phase reactants such as C-reactive protein (CRP) and fibrinogen. Both of these biomarkers are not only indicators of inflammation but also active contributors to the pathophysiological processes underlying atherosclerosis and atherothrombosis (Delange et al., 2018).

In a case-control study conducted by Widén et al., two cohorts—patients with acute coronary syndrome (ACS) and matched controls—were compared. The study revealed a significantly higher prevalence of periodontitis among ACS patients (52.6%) than among controls (12.8%). A strong association between periodontitis and ACS was reported, with an odds ratio of 7.5 (95% CI: 3.4–16.8, P < 0.001). Furthermore, ACS patients exhibited significantly elevated levels of IL-8 and vascular endothelial growth factor (VEGF) compared to controls; however, these elevations did not correlate with periodontal status (Anitha et al., 2013).

Matrix metalloproteinases (MMPs) are a group of inflammatory enzymes responsible for regulating connective tissue metabolism. Their upregulation in periodontitis lesions contributes to the degradation of collagen fibers within periodontal tissues. In cardiovascular disease, MMPs are similarly involved in atherogenesis and the destabilization of atheromatous plaques. Patients with chronic periodontitis demonstrate elevated levels of matrix metalloproteinase-8 (MMP-8) in gingival crevicular fluid, saliva, and serum compared to individuals with gingivitis or periodontally healthy controls (Delange et al., 2018).

Nitric oxide (NO), a key vasodilator synthesized by endothelial cells and platelets, plays a central role in maintaining endothelial function. Increased systemic oxidative stress, often a result of chronic inflammation, can reduce NO bioavailability, contributing to vascular dysfunction. Emerging evidence suggests that nonsurgical periodontal therapy enhances platelet-derived NO production via the L-arginine–NO–cyclic guanosine monophosphate (cGMP) pathway. While platelet aggregation remains unaffected, the post-treatment increase in NO bioavailability may enhance antioxidant defences and provide vascular protection against atherosclerosis (Siqueira et al., 2013; Pop et al., 2014; Pignatelli et al., 2020).

## 5 Atherosclerosis- pathophysiology and cardiovascular risk factors

The concept of the cardiovascular continuum describes a progressive chain of pathophysiological events initiated by various cardiovascular risk factors. If left untreated, these factors can lead to atherosclerosis, ischemic heart disease, and carotid artery stenosis. As the continuum advances, it may result in major complications such as acute myocardial infarction, stroke or heart failure (Pop et al., 2014). Atherosclerosis is the process that has long been described and has consistently remained an area of significant scientific interest. Despite extensive research, the pathophysiology of the atherosclerotic process remains more of a hypothesis than a clearly and precisely defined mechanism. Consequently, several hypotheses have been proposed regarding the development of atherosclerosis—some longstanding, others more recent—including thrombotic, inflammatory, dysmetabolic, and infectious mechanisms. In most cases, these complex pathways converge into the final phenotype of a multifactorial disease (Loesche et al., 1993).

Inflammatory cells—particularly foam cells and T lymphocytes—play a key role in the development and progression of atherosclerosis, being present even in the early stages of the disease. Injury to the intimal layer of the vessel wall facilitates the migration of smooth muscle cells from the media into the intima. Consequently, the atherosclerotic plaque consists of three major components: lipids, inflammatory cells, and smooth muscle cells, as well as connective matrix tissue, which may also contain thrombi at various stages of development (Rognoni et al., 2015).

The atherosclerotic plaque evolves through multiple stages, eventually progressing to a complicated plaque. However, all stages essentially represent an inflammatory response to initial vascular injury, mediated by cytokines. The most widely accepted hypothesis is that of repetitive injury and repair of the vascular intima (Boudoulas et al., 2016). Recent data suggest that inflammation and infection play a major role in the pathophysiology of atherosclerosis in the vascular wall and within atherosclerotic plaques (Visseren et al., 2021). Therefore, atherosclerosis can be defined as a chronic inflammatory disease with cyclical periods of activation, with patients exhibiting atherosclerotic plaques of varying severity, inflammatory activity, and composition.

Arterial hypertension, dyslipidemia, diabetes mellitus, and smoking are major risk factors for atherosclerosis. The above-mentioned risk factors, along with oxidative stressors (e.g., superoxide radicals) and angiotensin II, stimulate the production of adhesion molecules, pro-inflammatory cytokines, chemotactic proteins, and vasoconstrictors. In addition to traditional risk factors, hyperhomocysteinemia and hyperfibrinogenemia also promote atherosclerosis by contributing to endothelial injury (Dai et al., 2022).

The global reduction in cardiovascular mortality is largely attributed to population-level changes in cardiovascular risk factors. Recent data show that, in addition to age and other non-modifiable risk factors, arterial hypertension, unhealthy dietary habits, dyslipidaemia and systemic inflammation are among the most significant contributors to the progression of atherosclerosis (Dădârlat-Pop et al., 2020). Unfortunately, the prevalence of these risk factors has not significantly declined over the past two decades, despite sustained efforts to promote a healthy lifestyle. Moreover, elevated fasting blood glucose and increased body mass index have reached epidemic proportions worldwide (Yamawaki, 2011).

## 6 Inflammatory markers and risk of atherosclerosis

The stimulation of pro-inflammatory cytokine expression—such as IL-1, IL-6, and TNF-α—along with the proliferation of vascular smooth muscle cells lead to subsequent vascular dysfunction. Moreover, increased release of cellular adhesion molecules (VCAM-1, ICAM-1), and heightened oxidative stress through decreased endothelial nitric oxide synthase (eNOS) expression and subsequent reduction in nitric oxide (NO) are also important mechanisms in the development and progression of the atherosclerotic process.

Other several pro-inflammatory cytokines are known to contribute to the progression of the atherosclerotic plaque. These include leptin, TNF-α, IL-6, monocyte chemoattractant protein-1 (MCP-1), plasminogen activator inhibitor-1 (PAI-1), resistin, visfatin, and omentin. As previously mentioned, leptin, angiotensinogen, TNF-α, IL-6, MCP-1, PAI-1, resistin, and visfatin are closely associated with both inflammatory and atherosclerotic processes (Seropian et al., 2016).

Matrix Metalloproteinases (MMPs) are a family of zinc-containing endopeptidases involved in the degradation of the extracellular matrix. They also regulate the activity of various inflammatory cytokines, including TNF-α and IL-1β (Lubrano, 2015).

Lectin-like Oxidized Low-Density Lipoprotein Receptor-1 (LOX-1) is a receptor for oxidized LDL (ox-LDL), which forms under oxidative stress. LOX-1 plays a dual role: it facilitates the incorporation of lipid components into cells and contributes to atheroma plaque formation, thus increasing cardiovascular risk (Söder et al., 2005). Studies have shown a correlation between metabolic disorders and elevated circulating LOX-1 levels, as well as its early involvement in atheroma plaque development. Microparticles are released by activated endothelial cells, monocytes, and platelets. They exhibit pro-inflammatory effects, notably through IL-1 mediation (Jönsson et al., 2020).

## 7 Carotid artery disease and periodontal disease

There is data showing that periodontal disease is associated with a 19% increased risk of developing future coronary heart disease and stroke (Widén et al., 2016). Moreover, current evidence indicates a significant association between periodontal disease and the atherosclerotic process, even at its early and subclinical stages.

As previously discussed, periodontal disease is closely associated with cardiovascular disease, not only through the presence of common modifiable and non-modifiable risk factors—such as smoking, diabetes, obesity, and metabolic syndrome—but also through the systemic inflammatory response that characterizes both conditions. Chronic periodontitis leads to a persistent low-grade inflammatory state, marked by elevated levels of circulating pro-inflammatory cytokines, acute-phase reactants, and immune system activation, all of which play key roles in the development and progression of atherosclerosis.

Importantly, recent microbiological and molecular studies have provided compelling evidence that specific periodontal pathogens—including Porphyromonas gingivalis, Aggregatibacter actinomycetemcomitans, and Tannerella forsythia—can enter the bloodstream through ulcerated periodontal pockets and translocate to distant sites, including the vascular endothelium. These bacteria have been identified within atherosclerotic plaques suggesting a potential direct microbial contribution to to atherosclerotic plaque progression and instability (Mazzolai et al., 2024).

Thus, the association between periodontal and cardiovascular disease appears to be multifactorial, involving a complex interplay between systemic inflammation, shared risk factors, and direct microbial effects (**Figure 1**). This growing body of evidence supports the hypothesis that periodontitis may not only coexist with, but also potentially exacerbate, the atherosclerotic process. A recent study demonstrates an independent association between the severity of periodontal disease and carotid plaque burden, indicating that each additional periodontal pocket ≥4 mm corresponds to an estimated 0.34 mm² increase in carotid plaque area. Considering that individuals in the highest quartile presented with an average of 27 pockets—equivalent to roughly 9 mm² of additional plaque—and that even a 5 mm² increase in carotid plaque is linked to elevated cardiovascular risk, these findings underscore the need for intervention studies evaluating periodontal therapy as a strategy for cardiovascular disease prevention (Jönsson et al., 2020).

Carotid atherosclerotic plaque is defined as focal arterial wall thickening encroaching into the lumen by ≥ 0.5 mm or ≥ 50% of surrounding vessel according to Mannheim consensus, or as atherosclerotic thickening ≥1.5 mm or protuberant into the lumen, according to the American Society of Echocardiography (Mazzolai et al., 2024). Carotid artery stenosis is defined as ≥50% narrowing of the extracranial internal carotid artery, assessed by duplex ultrasound using the NASCET formula (Mazzolai et al., 2024).

Optimal medical management of asymptomatic carotid stenosis centers on the comprehensive correction of cardiovascular risk factors through both lifestyle modification and pharmacological therapy. The primary objective is to reduce the incidence of cerebrovascular events and overall cardiovascular morbidity (Mazzolai et al., 2024). Symptomatic carotid stenosis, as well as asymptomatic stenosis with high risk features associated with increased risk of stroke, require not only optimal medical therapy — typically including antithrombotic therapy and statins — but also revascularization, either through carotid endarterectomy or carotid artery stenting. The choice of intervention depends on factors such as vascular anatomy, lesion complexity, and, importantly, the patient’s preference, following a shared decision-making process (Noack et al., 2017).

To the best of our knowledge, there are few studies regarding the association between periodontitis and carotid artery disease. D. Jönsson et al. showed that there is an independent association between the prevalence and extent of periodontal disease and carotid plaque prevalence and severity (Jönsson et al., 2020). However, data in the current scientific literature remain limited regarding the association between the severity of periodontal disease and the prevalence of carotid plaques.

We have to take into consideration the findings of a study which indicate that periodontal disease is associated with early arterial structural changes, as reflected by mean intima-media thickness (mean-IMT), but does not show a significant relationship with maximal intima-media thickness (max-IMT), which is more closely linked to advanced atheroma formation. These results suggest that periodontal inflammation may contribute primarily to medial hypertrophy and vascular remodeling during the early stages of atherosclerosis rather than to the development of more advanced, plaque-related lesions. Given that previous investigations have examined mean-IMT and max-IMT separately in relation to periodontal status, the present study adds to the literature by evaluating both indices concurrently and demonstrating their differential associations. Further longitudinal research is warranted to elucidate the mechanisms underlying these relationships and to determine whether periodontal disease plays a causal role in the progression of subclinical atherosclerosis (Kida et al., 2023).

A recent systematic review reported that evidence supporting the secondary prevention of cardiovascular disease (CVD) in patients with periodontitis is unreliable, and that the available evidence for primary CVD prevention in this population remains very low in quality and inconclusive regarding the comparative effectiveness of scaling and root planning—with or without adjunctive antibiotics—versus supragingival scaling (Arbildo-Vega et al., 2024).

Demonstrating a direct causal relationship could serve as a foundation for preventing the progression of carotid atherosclerotic plaques and, consequently, reducing the risk of ischemic stroke. Studies have shown that measuring alveolar bone loss (ABL) on panoramic radiographs may serve as an effective method for identifying patients at increased risk of carotid artery calcification (CAC), with ROC analyses indicating that ABL is a superior screening indicator compared with the number of remaining teeth (Dewake et al., 2020).

In conclusion, periodontal disease and carotid artery disease appear to be interconnected through a series of shared pathophysiological mechanisms, including chronic systemic inflammation, immune activation, and the presence of common modifiable risk factors such as smoking, diabetes, and obesity. Moreover, the detection of periodontal pathogens within atherosclerotic plaques suggests a potential microbial contribution to vascular disease progression.

Despite these associations, current evidence remains insufficient to establish periodontal disease as an independent and direct risk factor for the development of carotid atherosclerotic plaques. The heterogeneity of study designs, populations, and diagnostic criteria in existing research limits the ability to draw definitive conclusions. Additionally, the potential confounding influence of traditional cardiovascular risk factors further complicates the interpretation of findings.

Therefore, well-designed, large-scale prospective studies are needed to clarify the causal relationship between periodontal disease severity and carotid plaque development and progression. Understanding this link more precisely could offer new insights into cardiovascular risk stratification and may support the integration of periodontal evaluation and management into broader strategies for stroke prevention. Therefore, the identification and monitoring of individuals at risk for carotid artery disease could be achieved through targeted screening. This approach may facilitate early access to cardiovascular evaluation and treatment, helping to prevent complications and improve long-term outcomes. Moreover, it supports optimal medical management by promoting interdisciplinary collaboration among dental professionals, primary care providers, and cardiovascular specialists.
